# Evaluating the Quality of Health Information in a Changing Digital Ecosystem

**DOI:** 10.2196/11129

**Published:** 2019-02-08

**Authors:** Alla Keselman, Catherine Arnott Smith, Anita C Murcko, David R Kaufman

**Affiliations:** 1 Division of Specialized Information Services National Library of Medicine National Institutes of Health Bethesda, MD United States; 2 University of Wisconsin-Madison Madison, WI United States; 3 Arizona State University Scottsdale, AZ United States

**Keywords:** eHealth, eHealth literacy, type 2 diabetes mellitus, consumer health information, health literacy, information evaluation, information quality, information literacy

## Abstract

**Background:**

Critical evaluation of online health information has always been central to consumer health informatics. However, with the emergence of new Web media platforms and the ubiquity of social media, the issue has taken on a new dimension and urgency. At the same time, many established existing information quality evaluation guidelines address information characteristics other than the content (eg, authority and currency), target information creators rather than users as their main audience, or do not address information presented via novel Web technologies.

**Objective:**

The aim of this formative study was to (1) develop a methodological approach for analyzing health-related Web pages and (2) apply it to a set of relevant Web pages.

**Methods:**

This qualitative study analyzed 25 type 2 diabetes pages, which were derived from the results of a Google search with the keywords “diabetes,” “reversal,” and “natural.” The coding scheme, developed via a combination of theory- and data-driven approaches, includes 5 categories from existing guidelines (resource type, information authority, validity of background information sources, objectivity, and currency) and 7 novel categories (treatment or reversal method, promises and certainty, criticisms of establishment, emotional appeal, vocabulary, rhetoric and presentation, and use of science in argumentation). The coding involves both categorical judgment and in-depth narrative characterization. On establishing satisfactory level of agreement on the narrative coding, the team coded the complete dataset of 25 pages.

**Results:**

The results set included “traditional” static pages, videos, and digitized versions of printed newspapers or magazine articles. Treatments proposed by the pages included a mixture of conventional evidence-based treatments (eg, healthy balanced diet exercise) and unconventional treatments (eg, dietary supplements, optimizing gut flora). Most pages either promised or strongly implied high likelihood of complete recovery. Pages varied greatly with respect to the authors’ stated background and credentials as well as the information sources they referenced or mentioned. The majority included criticisms of the traditional health care establishment. Many sold commercial products ranging from dietary supplements to books. The pages frequently used colloquial language. A significant number included emotional personal anecdotes, made positive mentions of the word *cure*, and included references to nature as a positive healing force. Most pages presented some biological explanations of their proposed treatments. Some of the explanations involved the level of complexity well beyond the level of an educated layperson.

**Conclusions:**

Both traditional and data-driven categories of codes used in this work yielded insights about the resources and highlighted challenges faced by their users. This exploratory study underscores the challenges of consumer health information seeking and the importance of developing support tools that would help users seek, evaluate, and analyze information in the changing digital ecosystem.

## Introduction

### Background

Evaluating the quality of online consumer health information has been a central issue in consumer health informatics for many years. However, with the emergence of new Web media platforms and the ubiquity of social media, the need for critical evaluation has taken on a new dimension and urgency. Individuals living with life-threatening and chronic diseases search the internet for treatment alternatives. Many such searches lead to sites containing nonevidence-based advice with targeted marketing and clickbait headlines.

Type 2 diabetes is a chronic disease that, according to the American Diabetes Association (ADA), affects 25 million Americans. Another 7 million may be undiagnosed, whereas approximately 80 million have impaired glucose tolerance, also known as *prediabetes*. These individuals have access to a plethora of online resources of highly variable quality. The objectives of this qualitative formative study are to develop a methodological approach for analyzing health-related Web pages likely to be viewed by individuals with type 2 diabetes and apply it to a set of relevant pages [1].

### Quality of Online Health Information

For health information seekers, the World Wide Web can be a source for both valuable information and misinformation. In the early 1990s, Gordon Guyatt was credited with coining the term *evidence-based medicine* or EBM, reflecting the hierarchy of scientific evidence employed in the development of clinical advice [2]. In 1997, MEDLINE indexed the first study evaluating consumer health Web pages [3]. Published in British Medical Journal, the study reviewed 41 pages with advice on managing children’s fever at home, concluding with an alarm that “only a few web sites provided complete and accurate information for this common and widely discussed condition.”

Today, the problem persists, and the rapidly growing popularity of social media is making the problem of identifying quality information more pressing. For example, although Facebook has recently made an effort to reduce unsolicited commercial content that appears as news headlines, these are not typically vetted, checked for accuracy, or monitored in any way [4,5]. Many sites prey on vulnerable populations who may be receptive to promises of a quick and easy cure or an alternative to medical establishment recommendations.

Health-related misinformation may be considered on a continuum that ranges from deliberately deceitful with the intent to promote specious products to sites that may more benignly endorse a product or claims that lack scientific credibility. The epidemic of fake or controversial health news presents formidable challenges for consumers and health educators. It also provides interesting research opportunities for the consumer health informatics community.

### Type 2 Diabetes Reversal as a Theme of Consumer-Targeting Websites

Type 2 diabetes is a serious disease that, over time, can damage small blood vessels and nerves, causing serious problems in the eyes, heart, brain, kidneys, skin, and feet, ranking in the top ten “killer” diseases in the United States [1]. This complex chronic disease starts with silent metabolic changes that precede symptoms and frank hyperglycemia (elevated blood glucose) by 7 to 10 years. With its ubiquitous and growing prevalence, type 2 diabetes is the focus of many consumer-targeting health information websites. A common theme is *reversal* of type 2 diabetes.

From the perspective of evidence-based medical authority (eg, as exemplified by the clinical practice recommendations of the ADA [6]), type 2 diabetes can be prevented, postponed, and placed into remission by lifestyle measures (diet, exercise, stress reduction) and therapy. However, except in rare and extreme circumstances, it cannot be cured or reversed. Medical literature references to diabetes *reversal* are sparse and limited to those on very low carbohydrate diets, postbariatric surgery, or in experimental animal models. Therefore, frank *reversal* of the complex metabolic derangements of type 2 diabetes is uncommon and quite difficult to accomplish. The lay use of the term *diabetes reversal* observed in the websites of interest, therefore, does not signal true medical reversals; instead, these are descriptions of diseases in remission characterized by reduction or discontinuation of medication. However, the ambiguity can lead readers to assume that reversal and cure are synonymous.

### Barriers to Effectively Negotiating the World of Digital eHealth

The danger of inaccurate health information is heightened by the public’s potential vulnerability to it. Factors that increase an individual’s vulnerability vary from a desperate desire for cure to dissatisfaction with traditional health care and limited health literacy. Health literacy can be defined as “the degree to which individuals have the capacity to obtain, process, and understand basic health information and services needed to make appropriate health decisions” [7]. The magnitude of the problem, affecting more than 90 million Americans, and its impact on health care and public health have been well established. Health literacy-related knowledge and skills are particularly deficient among vulnerable populations such as the elderly [8], disadvantaged youth [9], or people with lower levels of education. When individuals with lower health literacy conduct Web searches, they rely on criteria that do not correspond to the commonly cited quality guidelines when evaluating online health information [10]. For example, Mackert et al [11] showed that individuals low in health literacy used “position in search results, quality of pictures, celebrity endorsement, and website authorship as criteria to evaluate online health information.”

Newer concepts of health literacy frame it in terms of complex multidimensional models with broad applications in medicine and public health. For example, Osborne et al [12] used a validity-driven approach to develop a 9-factor Health Literacy Questionnaire (HLQ) model of health literacy. Examples of the model’s scales include abilities to establish relationships with health care providers, take responsibility for one’s own health, and obtain social support. Sørensen et al [13] developed an integrative conceptual model of health literacy drawing on a systematic literature review of 17 definitions and 12 conceptual models. This Health Literacy Survey (HLS) model integrates medical and public health as well as individual and population views of health literacy [14]. The new models underscore the complex array of individual and sociocultural factors underlying health information functioning.

Whereas health literacy enablers and barriers operate across a range of situations, electronic health (eHealth) literacy has emerged as both a distinct construct and an area of research into competencies needed for successful functioning in the world of digital health information [15]. Norman and Skinner introduced an influential eHealth literacy model, which comprised the following 6 types of literacy:

1. *Computer literacy*: the skills to use computers productively.
2. *Information literacy*: the skills to articulate information needs; to locate, evaluate, and use information; and to apply information to create and communicate knowledge.
3. *Media literacy*: the ability to select, interpret, evaluate, contextualize, and create meaning from resources presented in a variety of visual or audio forms [16].
4. *Conventional literacy and numeracy*: reading comprehension and quantitative skills for interpreting information artifacts such as graphs, scales, and forms.
5. *Scientific literacy*: familiarity with basic biological concepts and the scientific method as well as the ability to understand, evaluate, and interpret health research findings using appropriate scientific reasoning.
6. *Health Literacy*: the acquisition, evaluation, and appropriate application of relevant health information as described previously.

Chan and Kaufman extended and applied this model to analyze how individuals use Web resources to answer questions across different health topics [17-19]. They noted that the most frequently encountered barriers related to information literacy. These resulted in failures to identify relevant links and cues on websites, locate relevant information, and evaluate the trustworthiness and credibility of health information [9,20,21]. On the other hand, the skills associated with media literacy, especially in the context of new media or social media are not well understood [22-24]. Similarly, health information studies typically do not address scientific literacy.

Scientific literacy, as it pertains to matters of health, has been an area of inquiry in educational research for some time [25]. The concept extends beyond knowledge of specific scientific concepts, also involving knowledge *about* science and attitudes *toward* science. Knowledge about science is essential for guiding individuals in recognizing questions that are suitable for scientific investigation (eg, what treatment works best for a given condition) as well as important features of investigations (eg, randomization, control, sample size). Understanding what science is about, referred to as *the nature of science*, and positive attitude toward science also lead laypeople to differentiate between theory and evidence, favor systematic evidence as a source of knowledge, understand possible causes of scientific controversy, and appreciate the importance of logical consistency in explanations [26]. Although scientific literacy competencies used in this broad definition are relevant to health information seeking and evaluation, the concept has not been given much attention in consumer health research.

While health literacy is often discussed in research as a characteristic of individuals, HLQ and HLS perspectives suggest that it can also be conceived as characterizing the relationship between an individual and a set of resources. For example, different ways of presenting numerical data or scientific explanations may impact a reader’s ability to evaluate information, intentionally or unintentionally, for better or worse.

### Information Quality Evaluation Guidelines

Concern over health information quality online has been present from the dawn of the World Wide Web era. The Health on the Net Foundation’s code of conduct, launched in 1996, offered a set of best practice guidelines for website maintainers to follow [27]. To evaluate content itself, the DISCERN instrument was developed in 1996 and 1997 as a joint collaboration between the National Health Service and the British Library. DISCERN was a product of stakeholders chosen from across health care: generalist and specialist physicians, but also librarians and health communications specialists, self-help patient group representatives, medical publishers and journalists, and health services researchers [28].

DISCERN’s creators designed it to support websites’ evaluation by health information providers, serve as a checklist for content creators and a training tool for health care professionals, and most important, as a decision support for consumers who want to know more about a treatment they are using [29,30]. Although DISCERN was originally designed to target paper-based patient-facing leaflets, it can be used to evaluate any text-based information pertaining to treatment. This freely available instrument measures 16 items pertaining to markers of information quality (eg, reliability, relevance, balance, description of a treatment’s risks and benefits). Today this Web 1.0 tool remains in use, with results reported in over 150 published studies suggesting that DISCERN rankings are similar regardless of whether they are given by patients or consumers, or health care professionals.

### Study Objectives

This study aims to characterize health information sources in our ever-expanding digital ecosystem, including nonevidence-based pages presenting information about type 2 diabetes reversal. Specific objectives involve (1) reviewing top results pages in response to query about natural reversal of type 2 diabetes, (2) developing a methodological approach for capturing their essential content and informational characteristics, and (3) evaluating the utility of the approach with the above set of pages.

## Methods

### Page Selection

We started with reviewing a collection of non-EBM type 2 diabetes health advice Web pages that we have accumulated over the years of exploratory interest in the topic, noting frequent references to *reversal* and *natural* remedies. We then performed a search on Google using Firefox 61.0.2 browser on the first author’s machine using the keywords *diabetes*, *reversal*, and *natural* and collected the first 3 pages of results (31 pages). After reviewing these links, we excluded those that did not pertain to type 2 diabetes, focused on animals, or required creating a password-protected log-in. This resulted in 24 pages. The 25th page, hyperlinked from one of the search results, came from our initial review cluster.

### Coding Scheme Development and the Final Scheme

The coding scheme was developed using a combination of theory-driven and data-driven approaches [31]. First, the authors reviewed existing core information evaluation principles and criteria underlying various information science instruments, including the DISCERN instrument described above. The authors also attempted to code the pages using DISCERN, but found that it could not be justly applied across diverse information formats that included newspaper articles and patient testimonials. Afterwards, the authors conducted several rounds of review of 3 pages from the study set, noting and discussing perceived relevant characteristics. This resulted in the final coding scheme that included both pre-established categories (ie, represented in existing guidelines) and novel categories, as described in Table 1.

Our aim was to conduct detailed descriptive analysis that frames the user’s information-seeking experience. Although most questions are phrased to require binary (yes or no) responses, coding also involved writing short narrative responses (eg, “although citations are not provided, studies are described with partial information that would enables studies to be found eventually”). Three team members coded the data. To establish qualitative intercoder agreement [32,33], 13 pages were reviewed by 2 coders (in different permutations), which was followed by iterative in-depth team review and discussion of the coding. While the narrative data were not amenable to inferential statistical analysis, the team found the degree of narrative agreement [33] satisfactory; disagreements were resolved via discussion and the narratives were merged. The remaining pages were each reviewed by 1 coder (AK). With the exception of the *information authority* and *objectivity* coding that involved reviewing the parent site’s “About” page, all the coding was done based on the information within the page only. The pages were coded during July to November 2017, as they appeared at the time.

**Table 1:** Final coding scheme.

Source and category		Subcategories
**Existing guidelines**		
	Resource type	Digitized content (simultaneously published in traditional mass media) Static Web pages Web 2.0 content (Wikipedia, blogs, support groups, online communities, listservs, social networking sites, RSS feeds, and YouTube videos)
	Information authority	Is it clear who is responsible for the contents of the page? What are the author’s academic or professional credentials? Is there a way of verifying the legitimacy of the organization, group, company, or individual authoring the content? Is there any indication of the author's qualifications for writing on a particular topic? Is there a sponsoring or hosting organization that is separate from the author?
	Validity of background information sources	Is there content that needs to be cited, but is not? Are the sources for factual information clearly listed or cited so they can be verified in another source? (subsumes: are authors of testimonials and stated support verifiable?) Is the information from sources known to be reliable? Do citations or references actually support the information presented on the page? Are there endorsements by celebrity nonexperts? Who is referred to as “Dr” or “physician”? Is there mention of “secret recipe” (“virtually unknown method”) known only to the page’s owners or promoters? Is there a disclaimer on the page (what does it state)?
	Objectivity	Does the content appear to contain any evidence of bias? Is the page selling a product? Does the page encourage a certain action? Does the page-supporting organization engage in lobbying or advocacy or encourage lobbying or advocacy? Is there a link to a page describing the goals or purpose of the sponsoring organization or company? If there is any advertising on the page, is it clearly differentiated from the informational content?
	Currency	Are there dates on the page to indicate when the page was written, when the page was first placed on the Web, or when the page was last revised?
	Emotional appeal	Does the page contain emotional testimonies or personal anecdotes? Does the page contain disturbing photos or images of health care professionals and procedures?
**Data-driven**		
	Treatment or reversal method	What is the proposed diabetes treatment or reversal method?
	Promises and certainty	Does the page make a claim of having a solution (approach or product) producing results that are: Quick Painless or noninvasive or implemented via a simple procedure or with simple ingredients Relatively inexpensive Is there a promise of complete recovery for a condition that is known to be chronic or incurable?
	Criticisms of establishment	Is there implication or statement of conspiracy or purposeful misleading on the part of: Pharmaceutical companies? Doctors or conventional health care providers? Government agencies? Are there suggestions of media bias in covering relevant health issues? Are there implications or statements that the reader’s or viewer’s doctor is incompetent? Are there criticisms of biomedical research supporting the establishments’ guidelines? (eg, methodology and research focus because of funding).
	Vocabulary	Does the page refer to *cure* or other words that are unlikely to be used in evidence-based medical literature (eg, *proven*)?
	Rhetoric and presentation	Are there cliff-hangers in the content? For example, “In the next few minutes, I’m going to share with you the little-known natural remedy that will help you leave pills and needles behind forever...” Is there an appeal to buy something right away? Is the language very colloquial? Is there a long speech that culminates in a request for money?
	Use of science in argumentation	Are biological mechanisms of diseases and treatments presented? Are there claims that the coder perceives as: Exaggerated? False? Unverifiable? Mentioning controversial or not quite scientific concepts? Otherwise problematic? Is there a contrast between claims about the complexity or uncertainty of the condition or treatment and the simplicity and certainty of the proposed solution? (statements that are too good to be true?)

## Results

### Types of Web-Based Information Resources

Of the 25 pages, 13 were “traditional” static pages, 8 were Web 2.0 sources or static pages with video components. Four pages were digitized versions of printed newspaper or magazine articles. Three were patient testimonials.

### Diabetes Treatment or Reversal Methods

The number of remedies proposed by each page ranged from 1 to 7, with the mean of 2.24 (Table 2). The most commonly mentioned remedy involved taking dietary supplements. Recommended supplements included a range of herbs, vitamins, and minerals, with several mentions of cinnamon, turmeric, and chromium picolinate. These recommendations conflict with the ADA statement that “research has not been able to prove that dietary or herbal supplements (including omega-3 supplements, cinnamon, and other herbs) help to manage diabetes” [34]. The second most common recommendation was adhering to some general nutritional guidelines or healthy eating. General nutritional guidelines typically mentioned avoiding refined sugars and grains and eating more fiber and healthy fats. One page stated that a vegan diet was essential, describing an uncited study linking diabetes risk with consuming animal products.

Several pages promoted “superfoods.” Unlike general healthy eating guidelines, *superfoods* recommendations focused on specific “healing properties” of a particular food (eg, grapefruit). A special nutritional protocol advertised by one of the pages promised “to kill the microbes and parasites (eg, pancreatic flukes) identified by the consultation.” The undescribed protocol, apparently available from clinics promoted by the page, had to be followed by *electromedicine* said to “use gentle electrical waves to do the things necessary to rebuild the immune system.”

### Promises and Certainty

The specific promises made by the pages varied greatly. Fourteen of 25 pages either promised complete recovery or strongly implied that it was highly possible. In doing so, they often referred to *reversing* diabetes (eg, “type 2 diabetes is almost always reversible and this is almost ridiculously easy to prove”). Some expressed very high level of certainty: “If you follow our recommendations to the letter we guarantee that you will eventually be able to throw your medication away and never need it again!” Only 1 article, authored by a registered dietitian, discussed what it meant by *diabetes reversal*, explaining how *remission* is a more accurate term than *cure*.

Pages often promoted their approaches to “reversing” diabetes as quick, easy, and low cost. For example, 11 pages claimed to have a solution guaranteed to work within a specified period, from 11 days to 3 months. While some articles described difficult, extremely low-calorie regimens, 10 touted the ease of reversing diabetes (eg, “ridiculously simple”). Finally, 9 stressed that the treatments they proposed were inexpensive (“so inexpensive it might as well be free”).

**Table 2:** Numbers of pages (N=25) proposing specific remedies.

Remedy	Pages mentioning the remedy, n (%)
Supplements	14 (56)
General nutrition guidelines or healthy eating	13 (52)
Exercise	8 (32)
Special nutritional protocol: specific *superfoods*	7 (28)
Caloric reduction or intermittent fasting	5 (20)
Stress reduction	3 (12)
Improved sleep	2 (8)
Electromedicine	1 (4)
Optimizing gut flora	1 (4)
Weight loss	1 (4)

### Information Authority

Key information authority characteristics are summarized in Table 3. While 7 pages were authored or verified by physicians, other authors self-reported a range of qualifications for addressing the topic. Two authors were naturopaths. Several pages were written by journalists or patients giving testimonials. Of the remaining authors, 1 was a registered dietitian, 1 a self-described “clinical nutritionist” (certification not stated), and 1 an “ex-pharmaceutical chemist.” In addition, 4 authors were identified as health coaches or health experts without listed medical credentials. For example, 1, a former professional athlete, self-described as “one of the most trusted health and fitness experts.” Another self-identified as a health coach and a popular health and lifestyle reporter. However, another was described as “a catalyst voice” for alternative treatments and a founder of an independent health research foundation. Finally, 2 of the stated authors emphasized their lay relationship to the content, referring to themselves as average folk or concerned parents.

### Validity of Background Information Sources

The 25 sources varied in how they cited and validated the information they presented. As standards of providing citations differ greatly across various information types, it is not surprising that 20 pages contained uncited mentions of studies and data that could not be easily found based on those mentions. For example, phrases such as “studies show” were made without references or hyperlinked pointers to the studies. A representative example is the unreferenced statement that “a number of clinical studies have been carried out in recent years that show potential links between herbal therapies and improved blood glucose control.” Sources also made unreferenced statements that are not currently endorsed by leading relevant authority such as the ADA. For example, 1 page stated that “the solution to curing type 2 diabetes lies with killing the microbes and parasites inside the organs,” and that diabetes can be caused by “hepatitis c virus.” Another claimed that “cinnamon can curb the current epidemic of type 2 diabetes,” without references to any existing studies.

Sixteen out of 25 pages explained some of their information sources, either in the form of citations and references or by providing enough descriptive details so that the sources could be located with relative ease. Of these, 12 included sources that were deemed to be authoritative or reliable (eg, ADA, National Institute of Diabetes and Digestive and Kidney Diseases, and publications in journals listed in MEDLINE). When authoritative or reliable sources were included, they were used to support the specific statements to which they were linked in all but 1 case. Three of the pages not explaining their background sources claimed to have some sort of secret or “virtually unknown” recipe for treating diabetes.

### Objectivity

All pages were in the dot-com domain. Nineteen included a link to a page describing the site’s or the sponsoring organization’s goal. These goals varied in specificity, but typically had to do with information provision. Only 1 page called for legislative advocacy and encouraged readers to take actions such as petitioning the Department of Veteran’s Affairs to “employ licensed naturopathic physicians.”

Ten sites sold products ranging from alternative treatments and supplements to books and films. One had a paid access section. Some others did not sell products directly but contained links to the author’s books for sale or fee-for-service practice. Fourteen pages contained advertisements, which, in 4 cases, were not clearly differentiated from the page’s content.

### Currency

In 21 out of 25 cases, the page included a date indicating when the page was written, the information was first placed on it, or the page was copyrighted.

### Criticisms of the Establishment

Of the 25 sources, 14 made critical remarks about the pharmaceutical and health care establishment. Of these, 13 suggested malevolent intent or conspiracy on the part of various establishment agents (Table 4). Pharmaceutical companies received the greatest amount of criticism. For example, 1 page stated that “the pharmaceutical industry is a gigantic machine which has to sustain itself” and asked, “why would these companies be at all interested in truly reversing diabetes? How would that benefit them financially?” Another wrote, “Most big pharma companies don't know squat about how to reverse your diabetes.”

**Table 3:** The pages’ information authority characteristics (N=25).

Information authority characteristic	Pages, n (%)
Content has identifiable author(s)	22 (88)
Existence and legitimacy (accuracy of self-identification) of the author verified^a^	17 (68)
Sponsoring or hosting organization separate from the author^b^	11 (44)
Content authored or verified by someone described as a credentialed physician^c^	7 (28)

^a^As evidenced by a detailed on-site biography and/or external Web presence (eg, profiles in LinkedIn and online directories and business listings).

^b^For example, a newspaper or magazine, an association, and a public television channel.

^c^Stated MD (Medical Doctor) or DO (Doctor of Osteopathy) degree.

**Table 4:** Alleged malevolent intent or conspiracy agents (N=25).

Malevolent intent or conspiracy agents	Pages, n (%)
Pharmaceutical companies	7 (28)
Doctors	5 (20)
Biomedical research	3 (12)
Media	1 (4)
Other or unspecified	6 (24)

Two pages, 1 with a disclaimer, made explicit claims that diabetes medications recommended by health professionals are extremely dangerous and should be avoided (eg, “Avoid these 3 doctor recommended treatments: oral medication, insulin therapy, [and] other injectables”). One page suggested that the ADA dietary guidelines “seem to serve the medical practitioners more than the patients.” Another criticism warned readers that they had been “lied to for years.” In additions to claims of malevolence, 5 pages suggested medical doctors’ incompetence in diabetes management because of their lack of familiarity with nutrition and herbal medicine (eg, “doctors get little if any formal nutrition training in medical school”).

### Emotional Appeal, Vocabulary, Rhetoric, and Presentation

Fourteen pages included language that was judged by the coders as *very colloquial*, appearing to aim for establishing commonalities and rapport with the reader or viewer. For example, 1 page stated, “First I want you to know that here at [Company Name] we're really a lot like you. Average people who just so happened to be committed to helping people.” Several pages included emotional personal anecdotes such as stories of family members suffering from diabetes complications. As *natural* was part of the query used to identify pages for the study, it is not surprising that positive mentions of *natural remedies* and *natural treatment* were common. Fourteen pages included these terms; 10 made positive references to *cure*. Pages referred to nature and “Mother Nature” as a wise positive “healing” force, regaling the reader with “natural remedies,” “natural healing,” “natural processes,” and “natural cures.” Three pages (videos) included the infomercial approach of a long speech culminating in a direct request for an immediate purchase.

### Use of Science in Argument—Explanatory Mechanisms Behind Treatments

Of the 25 pages, 22 presented some biological explanations of their proposed treatment mechanism(s), with most pages including more than one. Often, pages combined mechanisms and methods widely accepted in standard care (eg, described by the ADA) with more uncertain and controversial ones. Depth of explanations ranged widely, from simple statements that a specific method (eg, a supplement) “improves sugar metabolism” to detailed explanations of intracellular molecular mechanisms, in our view, well beyond the level of comprehension of an educated layperson (eg, “Acetic acid protects the liver by increasing tolerance of lipogenesis and fatty acid synthesis responsible for improving cholesterol levels”).

The following explanatory mechanisms were particularly prominent:

- “*Unclogging” liver and pancreas for normal insulin production*: A number of pages recommending low-calorie diets or intermittent fasting explained that this method “unclogs” fat from liver and pancreas, thus restoring them to normal functioning essential for insulin production and glycemic control.
- *Reducing blood glucose and improving glucose metabolism (without mentioning insulin)*: Many pages explained their treatment methods (eg, specific foods and supplements) by stating that these methods “reduce[d] blood glucose,” “improve[d] glucose tolerance factor,” or “help[ed] metabolize glucose,” without mentioning insulin. Three pages that promoted exercise mentioned that it builds muscle that burns more glucose. In addition, a number of pages included a biological explanation of how foods high in sugar or refined carbohydrates created spikes in blood glucose (sugar) levels and needed to be avoided. These pages also often explained that foods high in fiber were beneficial because they slowed down glucose absorption.
- *Improving insulin secretion and insulin sensitivity*: A number of pages stated that foods or supplements implicated in their treatment methods influenced production or secretion of insulin, improving insulin resistance or sensitivity, and, in some cases, “mimicked insulin.”
- *Reducing inflammation*: Several pages related diabetes to inflammation, stating that food or supplements described by them fought it or increased “good bacteria” in the intestinal lining.
- *Strengthening cells and organs*: Several pages related diabetes to weakened immune system, “weakened organs” (in particular, liver and pancreas), and “weakened cells.” They proposed that their methods “strengthened” cells and organs. Mechanisms ranged from supplements that “help strengthen the cellular signal” to “electrical waves” that “kill parasites and microbes that weaken organs.”

## Discussion

Web pages about natural treatment of diabetes analyzed in this study proposed a number of reversal methods and differed greatly in terms of their alignment with accepted standard of care recommendations, promises, levels of certainty, authors’ background, transparency of sources, rhetoric, style, and attitude toward pharmaceutical and medical establishment. This exploratory study only looked at a small sample of pages pertaining to a single topic collected from 1 search on 1 machine. However, it underscores the challenges of consumer health information seeking and the importance of developing support tools that would help users seek, evaluate, and analyze information in the changing digital ecosystem. Future work within this research program will focus on extending the approach to a number of domains, developing more robust evaluation criteria, and exploring computational approaches to pages’ analysis.

### Is the Consumer Health Web Universe a Dangerous Place?

Consequences of following recommendations promoted by the sources analyzed in this study are likely to vary. The core lifestyle modifications recommended by many pages, namely, exercise, weight loss, stress reduction, and a healthy balanced diet are evidence-based components of conventional type 2 diabetes regimes. Very low–calorie diets and intermittent fasting may be beneficial, but because of the known risks (including death), they require medical supervision, especially for those with diabetes requiring medication. Supplements (including cinnamon), superfoods, and optimization of gut flora, promoted by many pages, have a low health risk but little-to-no proven benefit and can be financially draining. However, as illustrated in Table 2, most pages repackage the core lifestyle recommendations and add-on *essential* product purchases, muddying the water. The stakes for harm are even higher when pages promising to reverse diabetes undermine the use of medications, including recommending unsupervised medication discontinuation, and promote an antiscience attitude.

### Fit With Existing and Added Health Information Evaluation Criteria

One of the objectives of this work was to develop a methodological approach for analyzing the digital health information sources in the era of online videos and social media. The study suggests that both traditional and data-driven categories of codes (see Table 1) yielded insights about the resources and highlighted challenges faced by their users.

#### Existing Evaluation Criteria Categories

##### Information Authority

Assessing authority of information authors and sponsors was straightforward, except for the case of determining the ownership of a YouTube channel. In the majority of cases, pages analyzed in this study had clear authorship indicators, with the authors having sufficient Web presence to lend credibility to their stated identity and credentials. While a sizable minority of pages (7 of 25) was authored or verified by credentialed physicians, the majority were created by noncredentialed individuals.

##### Validity of Background Information Sources

Pages in this sample frequently described or mentioned scientific studies without providing references that would allow their unambiguous identification. They also typically did not reference their biological explanations or statements about treatments that were not aligned with ADA guidelines, developed upon an extensive review of the scientific evidence from peer-reviewed sources [35]. However, the same pages typically included citations of some external sources with background information about type 2 diabetes. These were often high-quality authoritative sources.

While assessing validity of cited information sources was straightforward, the expression of this criterion varied for different publication formats because of their differing conventions. For example, bibliography style references are difficult to present in videos and uncommon in newspaper articles where a detailed description of a background study is a more likely quality indicator. Still, the criterion of validity remains highly relevant and the lack of credible citations or pointers, or a mismatch between citations and their purported claims, raises concerns about information quality.

##### Objectivity

Across the range of information source types, this criterion was unambiguous. Page’s or sponsor’s goals, typically stated on the site’s About page, as well as the information about sales of relevant products and services, provided information helpful for judging objectivity. Selling services and products such as supplements by default indicated their endorsement.

##### Information Currency

Currency turned out to be a challenging criterion because of the range of events that could be time-stamped on the pages. Although most of the pages had a time stamp, these were more likely to be the dates of the page’s copyright than of information authorship.

#### Novel Evaluation Criteria Categories

##### Treatment or Reversal Method and Use of Science in Argumentation

These categories are discussed together because scientific argumentation usually explained the treatment methods. For this sample, the *use of science in argumentation* code proved valuable for elucidating the challenges facing health information seekers. The pages typically provided some biological information, claiming effects of substances or procedures on insulin production, glucose metabolism, and cells/organs/ microorganisms. Often, these explanations blended widely accepted biological mechanisms with controversial ones. While many pages limited their biology to simple causal statements such as “cinnamon improves insulin sensitivity,” others employed complexity well beyond the level of comprehension of an educated layperson. Many of the non-ADA-aligned treatment methods and biological mechanisms mentioned on the pages had corresponding coverage in peer-reviewed science literature, albeit scientific literature described them as more controversial, less certain, and limited to a narrow range of application (eg, demonstrated effects limited to animal studies or lacking adequate controls). Applying the *use of science in argumentation* code underscores the formidable challenge of supporting lay assessment of plausibility of online health information. It also suggests that the science literacy component of eHealth literacy deservers greater attention.

##### Promises

A promise of recovery from a chronic disease, with a high level of certainty and implied treatment simplicity, was a useful indicator of concern about a page’s quality. Promises were especially troubling when presented as guaranteed within a specific time frame.

##### Criticisms of Establishment

As a criterion, this one is easy to apply, revealing, and provides disconcerting information. The importance of the code is illustrated by the disturbing number of pages making critical comments in our sample (14 of 25). Criticisms of health care–related establishment are disconcerting because they attempt to discredit primary sources of evidence-based care, positioning nonevidence-based methods as primary, rather than complementary. In making critical claims, pages often had to tread the line between denouncing some official sources and yet conveying respect for science and evidence and support from some studies. The public’s response to the representation of science, doctors, and pharmaceutical organizations in the digital ecosystem merits further examination.

##### Emotional Appeal, Vocabulary, Rhetoric, and Presentation

General level of colloquialism and informality and the use of personal or emotional anecdotes seemed to be most related to document type, with more informal language used in videos and newspaper articles. Specific high certainty words such as *cure* and *guaranteed*, on the other hand, are potential quality signifiers that merit further research.

#### Scientific Literacy as a Dimension of eHealth Literacy Revisited

As mentioned earlier, this study underscores the importance of scientific literacy as a component of eHealth literacy. The role of science knowledge in daily life has long been debated in the fields of public health and science education. This study illustrates that scientific literacy, although important, should not be equated with content knowledge. It is often unrealistic to expect laypeople to have biomedical knowledge necessary to analyze the argument behind controversial treatment methods. Although some claims may be refuted by high school biology (eg, diabetes is a disease of “weak organs”), many remain difficult to evaluate even after a thorough analysis of a PubMed search. In such cases, the relevant aspects of science literacy are not specific content knowledge, but understanding the nature of science and scientific evidence, uncertainty, and the process of biomedical discovery. Such knowledge is likely to trigger skepticism about overgeneralizations, oversimplifications, and exaggerations inherent in many consumer-targeting pages that promise quick and easy fixes for complex health problems. Complexity of science literacy also underscores the importance of promoting traditional information evaluation criteria such as source authority and objectivity.

### Implications for Research and Practice: Ways to Support Researchers and Consumers

The methodological approach, described in this study, is a coding scheme designed expressly for research purposes, rather than as a tool for evaluating Web pages. Much more work, aimed at expanding, fine-tuning, simplifying, and validating the criteria, is needed before this approach can produce a numerical score that could be used to assess a Web page. Such a tool could be very valuable to both researchers and every day information seekers. It would be particularly beneficial if developed and validated for a wide range of online information sources, including blogs, message boards, videos, and other social media platforms.

In addition to providing an evaluation guide, medical and informatics organizations may investigate developing resources that address common controversial claims. The establishment often ignores nonevidence-based treatment recommendations, despite their visibility in the public domain. For example, the consumer portion of the ADA site dedicates very little space to a discussion about cinnamon. A thorough respectful explanation of why the use of cinnamon should be treated with caution may be a more effective way to help consumers. New models of health literacy, such as HLQ and HLS [12-14], that consider complex sociocultural determinants of information behaviors (eg, root causes of different attitudes toward medical establishment) could surface explanations as to why certain groups find specific messages to be compelling. It could also inform the presentation and content of explanations that counter potentially deleterious messages. Computation-based informatics tools may also play a role in helping users evaluate Web pages. This study suggests that certain terms and phrases, particularly those indicating high confidence and rejection of traditional medicine, may be alert markers. Research and development into automated language-based categorization may be useful in flagging suspect pages. Finally, this study suggests the importance of science education for the development of science literacy and the potential synergy between classroom science and health informatics.

## Abbreviations

ADA: American Diabetes Association
EBM: evidence-based medicine
eHealth: electronic health
HLQ: Health Literacy Questionnaire
HLS: Health Literacy Survey
